# Structural comparison of N-butyl-2-cyanoacrylate-Lipiodol (NL) and N-butyl-2-cyanoacrylate-Lipiodol-Ethanol (NLE) using scanning electron microscope

**DOI:** 10.1007/s11604-025-01751-3

**Published:** 2025-02-22

**Authors:** Yu Sasaki, Takuji Araki, Munetsugu Ban, Kodai Hujihara, Hiroto Imaimatsu, Hiroki Okada, Toshiyuki Oda, Hiroshi Onishi

**Affiliations:** 1 Department of Radiology, University of Yamanashi, 1110 Shimokato Chuo, Yamanashi, 409-3898 Japan; 2 Department of Anatomy and Structural Biology, University of Yamanashi, Yamanashi, Japan

**Keywords:** N-Butyl-2-Cyanoacrylate (NBCA), N-Butyl-2-Cyanoacrylate-Lipiodol-ethanol (NLE), Ethanol, Lipiodol, Scanning electron microscope

## Abstract

**Purpose:**

N-butyl-2-cyanoacrylate (NBCA) and Lipiodol mixture (NL) are widely used for emergency embolization due to their effective polymerization upon contact with blood. However, NBCA’s strong adhesive properties can cause complications, leading to the development of an NBCA-Lipiodol-ethanol mixture (NLE), which has shown reduced catheter adhesion. This study aimed to observe the structural differences between NL and NLE polymers using scanning electron microscopy.

**Materials and methods:**

Four different ratios of NBCA, Lipiodol, and ethanol (NLE230, NLE221, NLE150, and NLE141) were examined. The samples were injected into silicone tubes filled with human serum, and the polymerized specimens were collected and observed using scanning electron microscopy.

**Results:**

NLE230 formed a dense, three-dimensional honeycomb-like structure, whereas NLE221 exhibited a two-dimensional folded-sheet structure. Both NLE150 and NLE141 exhibited a folded-sheet structure; however, NLE141 was considerably more fragile, with cracks and rough surfaces, resulting in a structure that lacked uniformity.

**Conclusion:**

The differences in structure suggest that ethanol considerably influences the polymerization process. These differences may explain characteristics of NLE, such as low adhesion.

## Introduction

N-butyl-2-cyanoacrylate (Histoacryl; B. BRAUN, Melsungen, Germany) is a cyanoacrylate-based instant adhesive that forms a polymer following contact with blood. Owing to this characteristic, NBCA-Lipiodol mixture (NL) is used for emergency embolization of bleeding sites, including trauma-induced, obstetric, and gastrointestinal bleeding. NL is also widely applied to embolize arterial bleeds in patients with high coagulopathy [1–4]. However, NBCA may cause adhesion between the catheter and the vascular intima [5]. To overcome these problems, an NBCA-Lipiodol-ethanol mixture (NLE), which has been associated with less adhesion to the catheter than NL, has been developed [6]. The low adhesive strength of NLE allows it to be injected slowly and in large amounts without immediate catheter removal after NLE injection, and this characteristic is mainly utilized for embolization procedures in type 2 endoleaks, gastric varices, and arteriovenous malformations [7–9]. Previous studies have examined appropriate mixing ratios, indicating that if the proportion of NBCA falls below 30%, the polymer may become fragile, leading to the risk of fracturing and migration [10].

Despite its clinical use, observational reports on the polymer itself remain limited. While there have been reports on the staining and observation of thin sections of aneurysms embolized with NLE in swine [10], no reports have yet been published on the observation of the three-dimensional structure of the NLE polymer. Scanning electron microscopy (SEM) allows observation of non-thin sections. Using electrons, which have shorter wavelengths than light, an electron microscope can achieve a high resolution of 0.5–4 nm. SEM enables the observation of non-thin sections by irradiating a specimen coated with platinum or palladium and capturing reflected secondary electrons. Therefore, in this context, the present study aimed to observe the structural differences between NL and NLE using SEM.

## Materials and methods

The blood used in this study was exclusively the author’s own venous blood. The author has no underlying medical conditions or medications. Venous blood was drawn and left standing in a syringe for over six hours, after which the serum from the supernatant was collected and used as the sample. Each time sample was prepared; the same procedure was followed to ensure the use of fresh serum.

This is a self-experimentation study, and we obtained a certification from our institutional ethics committee confirming that no ethical application is required.

A silicone tube cut to 4 cm in length was filled with serum. For NL and NLE, four different mixtures of NBCA, Lipiodol, and ethanol were prepared in ratios of 2:3:0, 2:2:1, 1:5:0, and 1:4:1. These mixtures are referred to as NLE230, NLE221, NLE150, and NLE141, respectively. The proportion of NBCA was 40% in NLE230 and NLE221, and 16.7% in NLE150 and NLE141, respectively. Three sets of these compositions were prepared and labeled as follows: NLE230①, NLE230②, NLE230③, NLE221①, NLE221②….etc.

A scanning electron microscope (JSM-6510LV, JEOL, Japan) was used. To prepare the specimens, a 2.5 mL syringe with a three-way stopcock was used. To prepare the NLE, we first added NBCA and Lipiodol into a 2.5 ml syringe and mixed them 20 times. After that, we added ethanol and mixed them an additional 20 times. After which it was immediately injected into human serum using a 23G needle. NL and NLE mixtures were injected at 0.3 ml in each sample. To ensure the completion of the reaction, the sample was left undisturbed for 72 h. The silicone tube was then cut open to retrieve the polymerized specimen, which was washed with a surfactant, soaked in ethanol, and air-dried for degreasing and dehydration. The specimens were then cut, coated with Rh, and observed using SEM. The ion sputter used for Rh coating was E1030 (JEOL, Japan).

## Results

**Fig. 1:**
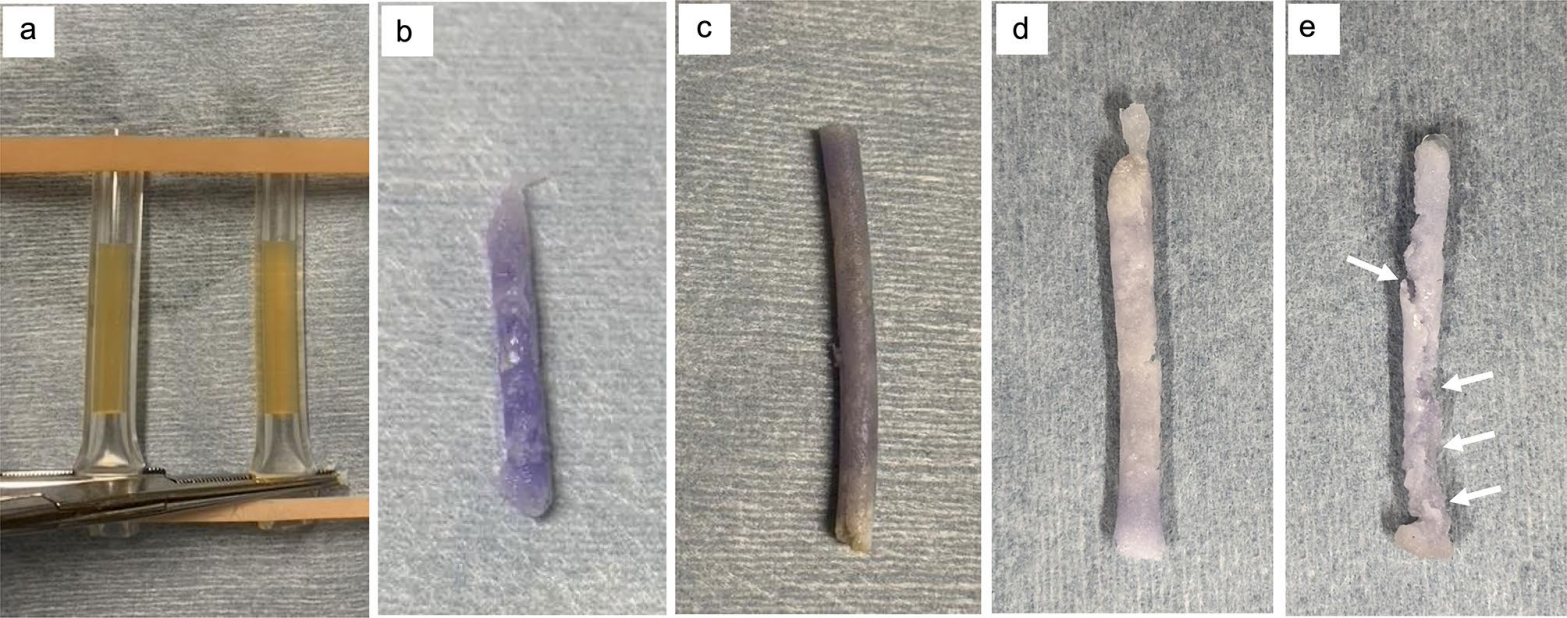
Sample preparation process. **a**: Silicone tube and human serum. The serum was the supernatant of venous blood after sedimentation. **b**: Polymerized sample of NLE230①. The surface was smooth, and the density was high. **c**: Polymerized sample of NLE221①. The surface was slightly rough, and the density was high. **d**: Polymerized sample of NLE150①. Although the density was low, it had a uniform structure. **e**: Polymerized sample of NLE141①. The polymer was fragile, with cracks present (white arrows), and the internal density was uneven

**Fig. 2:**
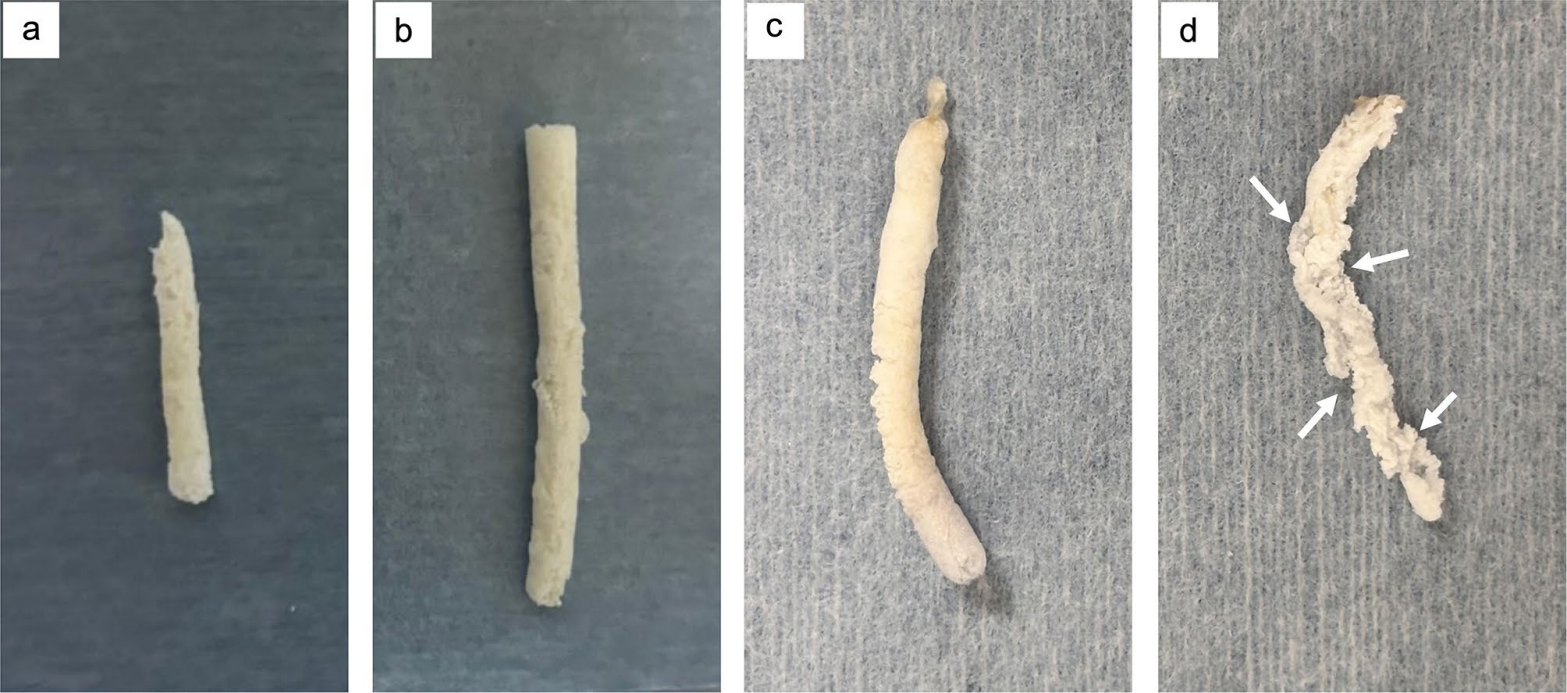
Samples after defatting and dehydration. **a**: Polymerized sample of NLE230①. The density was high, and the structure was preserved even after defatting and dehydration. **b**: Polymerized sample of NLE221①. The density was high, and no considerable changes were observed after washing. **c**: Polymerized sample of NLE150①. Although the density was low, there were no changes in the uniform structure. **d**: Polymerized sample of NLE141①. The polymer became more rugged during the washing process, with the cracks growing larger (white arrows). Some parts fragmented easily into small pieces, resulting in a highly brittle structure

When the mixture was injected into human serum, polymerization began immediately in all cases (Fig. 1). The polymerized product of NLE141 had a low density, was fragile, and fragmented during the degreasing and dehydration process with surfactant and ethanol (Fig. 2). Each polymerized specimen was cut into circular sections, coated with palladium, and observed using SEM.

**Fig. 3:**
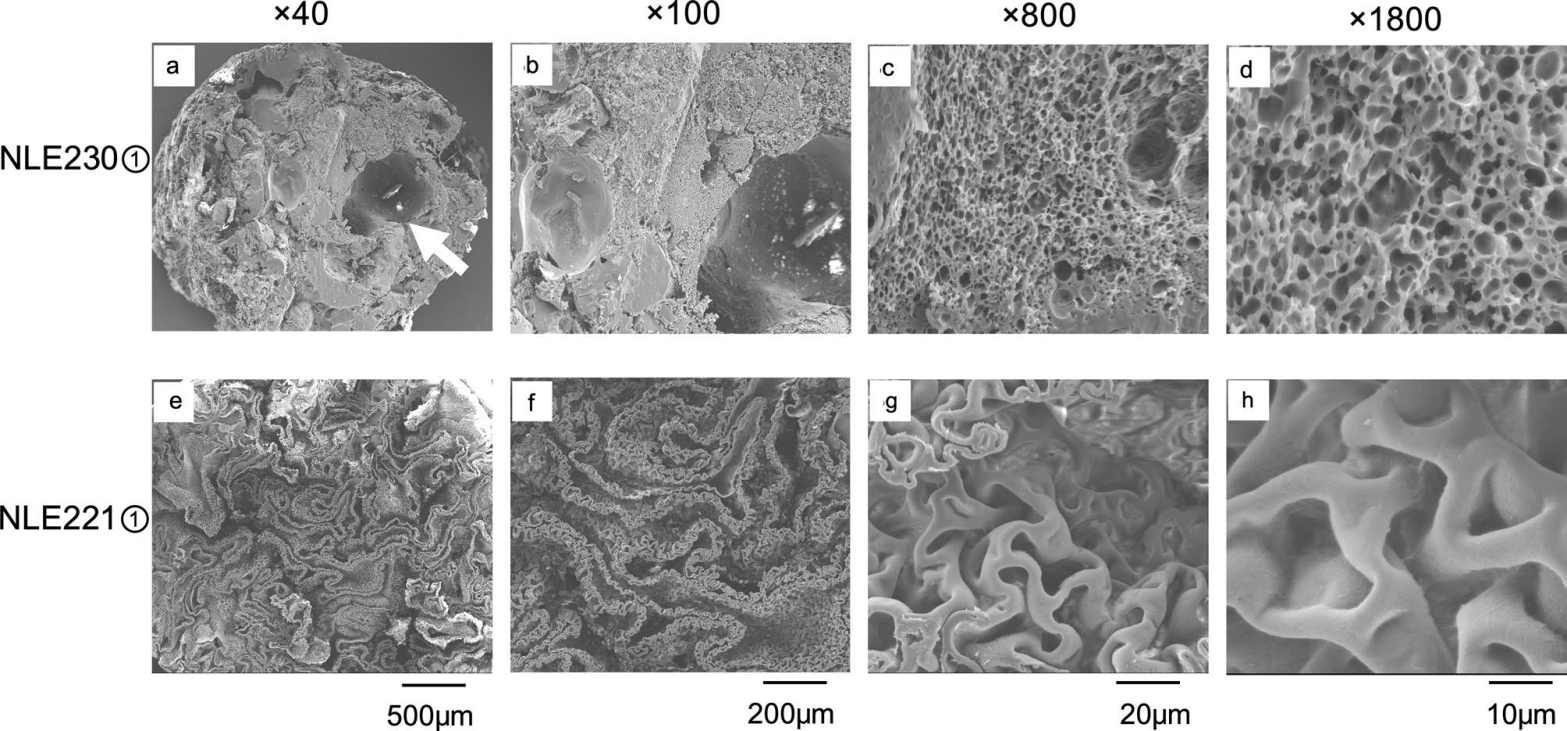
SEM analysis of NLE230① and NLE221①. The electron beam voltage during observation was 10 kV in all cases. **a**: NLE230① at 40 × magnification. Although large voids were present (white arrow), it exhibited high density. **b**: NLE230① at 100 × magnification. High density was observed, similar to that at 40 × magnification. **c**: NLE230① at 800 × magnification. The polymer contained numerous small voids. **d**: NLE230① at 1800 × magnification. The polymer had honeycomb-like structure, a high density and three-dimensional crosslinking, containing many tiny internal voids. **e**: NLE221① at 40 × magnification. Although the density was high, there were spaces present compared to NLE230①. **f**: NLE221① at 100 × magnification. The polymer had a loose structure resembling multiple layers of folded sheet. **g**: NLE221① at 800 × magnification. Small folded sheet were also observed on the surface of the large folded sheet. **h**: NLE221① at 1800 × magnification. Each crosslink was looser than that of NLE230①

**Fig. 4:**
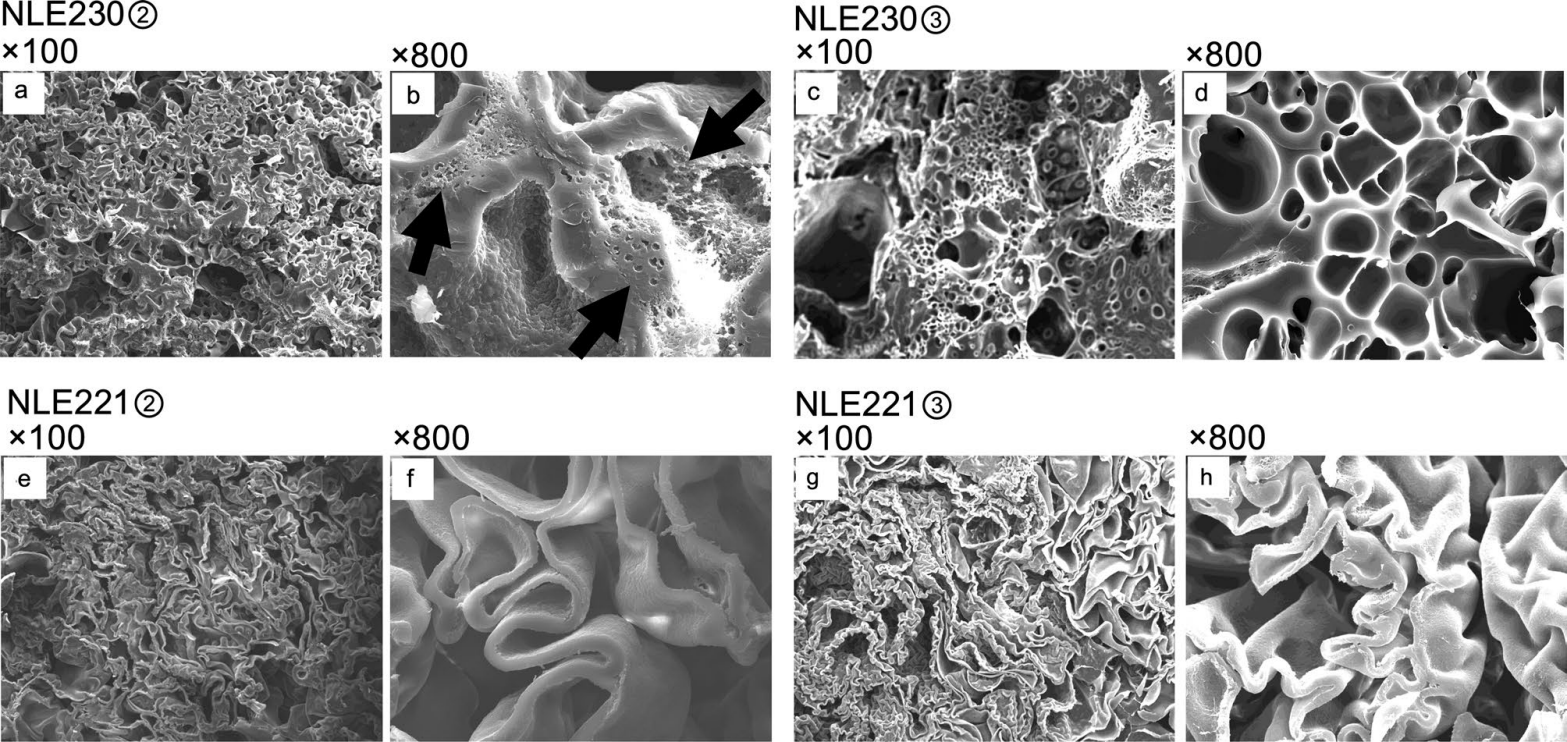
SEM analysis of NLE230②, NLE230③, NLE221②, and NLE221③. **a**: NLE230② at 100 × magnification. A folded-sheet structure was observed. **b**: NLE230② at 800 × magnification. Each sheet was thick, and regions with higher density honeycomb-like structure were also observed (black arrows). **c**: NLE230③ at 100 × magnification. Similar to NLE230①, it exhibited high density with both large and small voids. **d**: NLE230③ at 800 × magnification. Similar to NLE230①, a honeycomb-like structure was observed. **e**: NLE221② at 100 × magnification. Similar to NLE221①, a folded-sheet structure was observed. **f**: NLE221② at 800 × magnification. Each sheet was thinner compared to NLE230②. **g**: NLE221③ at 100 × magnification. Similar to NLE221① and NLE221②, a folded-sheet structure was observed. **h**: NLE221③ at 800 × magnification. The thickness of each sheet was also similar to that of NLE221① and NLE221②

NLE230① had a high polymer density, with some voids, and when magnified, it was observed to form a three-dimensional honeycomb-like crosslinked structure (Fig. 3). In contrast, NLE221② had a slightly lower density, resembling a two-dimensional folded-sheet structure similar to that of cardboard (Fig. 3). The same characteristics were observed for all three NLE221 and NLE230③ specimens. However, in the NLE230② sample, a structure was observed that appeared to be a mixture of both a honeycomb-like structure and a folded-sheet structure (Fig. 4).

**Fig. 5:**
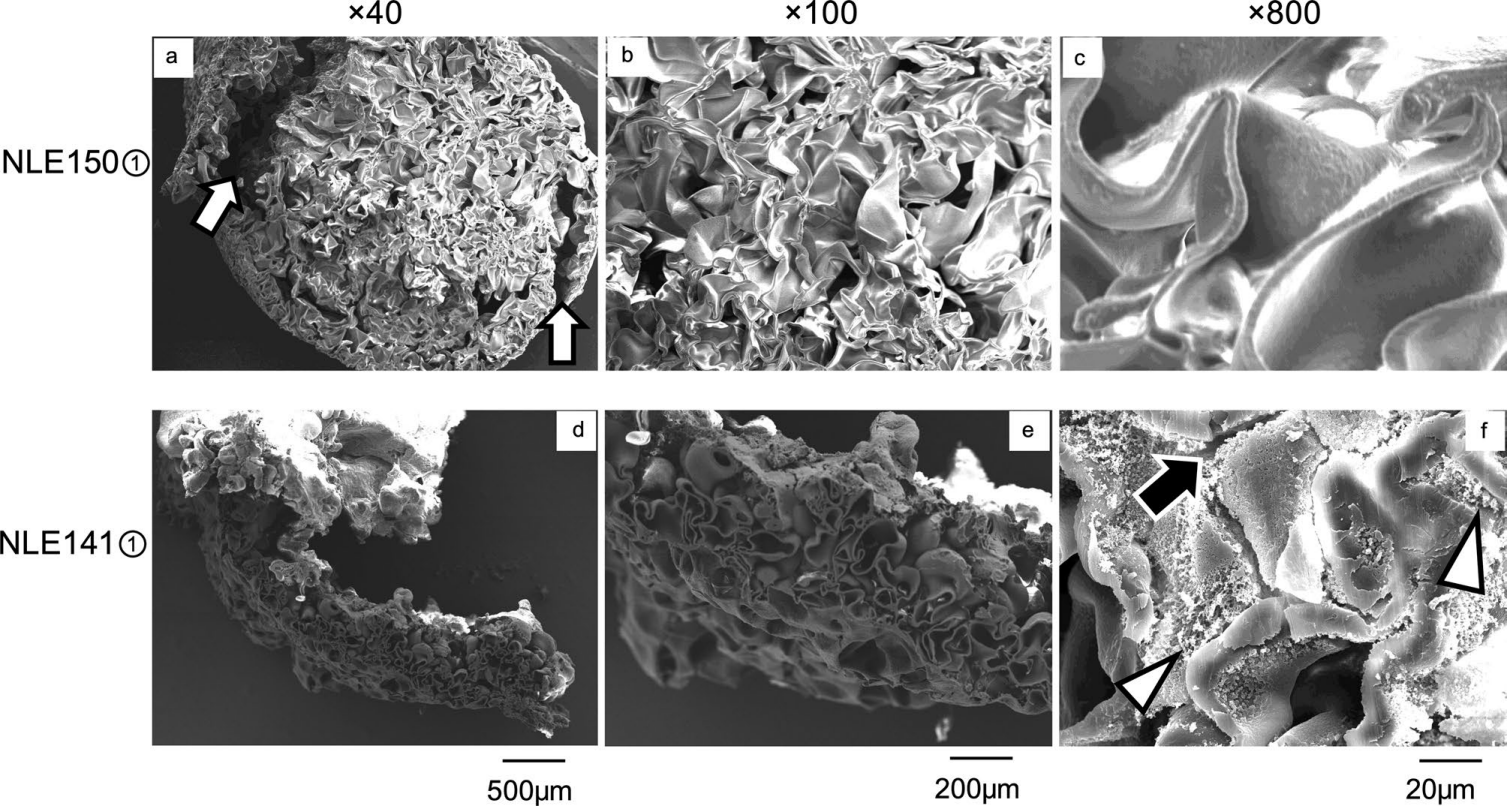
SEM analysis of NLE150① and NLE141①. **a**: NLE150① at 40 × magnification. It was composed of a folded-sheet structure, and in some areas, delamination was observed (white arrows). **b**: NLE150① at 100 × magnification. It exhibited folded-sheet structure in all areas. **c**: NLE150① at 800 × magnification. The sheet was thinner compared to NLE221 samples. **d**: NLE141① at 40 × magnification. The sample was fragile and easily fragmented into small pieces. The Rh coating on the brittle sample was uneven, resulting in a decrease in image quality. **e**: NLE141① at 100 × magnification. It had a combination of thin and wide rough sheets. **f**: NLE141① at 800 × magnification. It had some areas with a granular rough texture on the surface (white arrowheads), with some cracks observed in certain sections (black arrow)

NLE150① was composed of a folded-sheet structure, but compared to NLE221, the sheets were thinner and the gaps between them were more pronounced (Fig. 5). In some areas, delamination of the polymerized specimen was observed. NLE141① exhibited a structure predominantly composed of folded-sheet, similar to NLE221 (Fig. 5). However, even compared to NLE150①, the folding width of NLE141① was larger, and the density was lower. Additionally, the structure appeared fragmented in some areas, with noticeable cracks in the sheets and a rougher surface texture. Moreover, during electron beam irradiation in SEM observation, parts of the specimen fragmented and shifted, causing a reduction in image quality (Fig. 5).

**Fig. 6:**
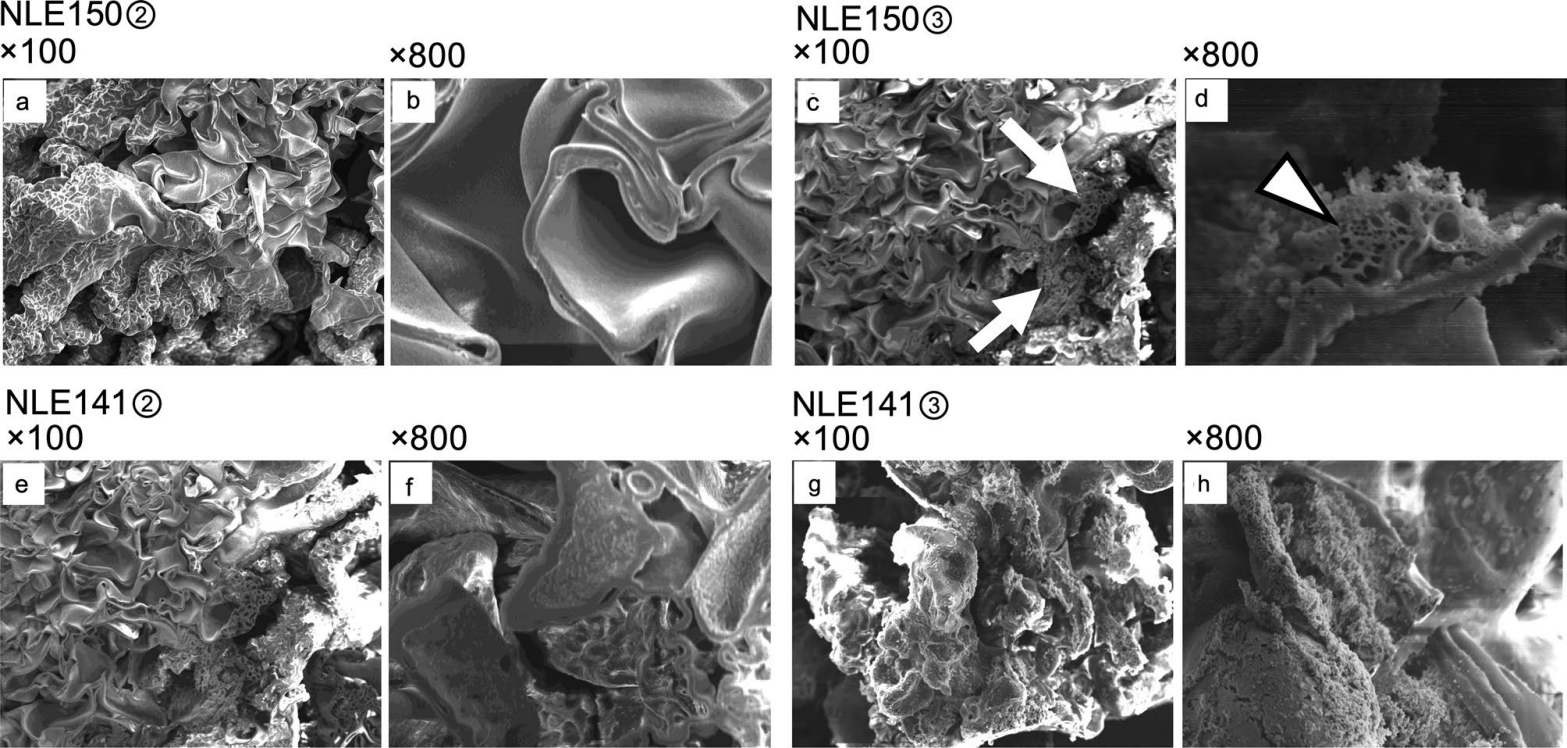
SEM analysis of NLE150②, NLE150③, NLE141②, and NLE141③. **a**: NLE150② at 100 × magnification. It was composed of a folded-sheet structure similar to NLE150①. **b**: NLE150② at 800 × magnification. The sheet was thin, similar to NLE150①. **c**: NLE150③ at 100 × magnification. It was composed of a folded-sheet structure with small uneven portions (white arrows). **d**: NLE150③ at 800 × magnification. In a small portion of NLE150③, a honeycomb-like structure was observed (white arrowhead). **e**: NLE141② at 100 × magnification. It had a combination of thin and wide rough sheets. **f**: NLE141② at 800 × magnification. Rough sheets were observed, similar to NLE141①. **g**: NLE141③ at 100 × magnification. The density was low, and the structure was uneven. **h**: NLE141③ at 800 × magnification. Rough sheets and irregular surface areas were observed, similar to NLE141① and NLE141②

In NLE150②③, the structure was generally consistent with that of NLE150①, with a small portion showing a honeycomb-like structure (Fig. 6). In NLE141②③, similar to NLE141①, a mix of folded-sheet structures and areas with cracks and rough surface irregularities was observed (Fig. 6).

## Discussion

Cyanoacrylate adhesives that contain NBCA react with nucleophiles and free radicals. Nucleophiles include anions, water molecules, and hydroxyl groups in alcohols, which can react with the covalent bonds of NBCA to initiate polymerization. However, the progress of polymerization varies depending on the reactivity of each nucleophile [11, 12].

Despite the reaction between ethanol and NBCA, three possible mechanisms ensure that NLE remains liquid until injected into the blood. First, the nucleophilicity of ethanol is extremely weak compared to anions such as Cl⁻ and HCO₃⁻ present in blood, resulting in a prolonged reaction. Secondly, the large amount of ethanol relative to NBCA can lead to infrequent contact between monomers, forming low-molecular-weight polymers that are less likely to precipitate as solids. Third, Lipiodol is known to slow the progression of polymerization when mixed with NBCA. Although polymerization occurs, it is prolonged, and the resulting polymers are very small, which could allow NLE to maintain a liquid-like state despite containing low-molecular-weight polymers until it is injected into the blood.

The characteristics of NLE include reduced adhesiveness and the fragility of the polymers [6]. One hypothesis is that if low-molecular-weight polymers are formed in NLE due to ethanol, when they react with blood, the fragmented polymers may not sufficiently adhere to catheters or blood vessels, leading to decreased adhesion. Additionally, due to the nature of these fragmented polymers, the strength of the polymerization may also be lower. To verify these hypotheses, it is necessary to observe and evaluate the NLE mixture before it comes into contact with blood. If we can measure the molecular weight of NL and NLE before injection into blood and polymerization occurs, it may provide evidence for the presence of low-molecular-weight polymers. Although there have been previous reports suggesting that NLE has weaker adhesiveness than NL in in vivo studies [7, 10], no research has been conducted to compare their adhesive strength in vitro. To test this hypothesis, it is necessary first to verify that NLE’s adhesiveness is indeed weaker than NL’s under in vitro conditions. This remains a challenge for future studies.

In this study, a honeycomb-like structure was observed in the NLE230 samples, whereas a folded-sheet structure was observed in the NLE221 samples. Previous report has suggested that adding ethanol to NL may accelerate the polymerization reaction [6]. The honeycomb-like structure was formed from tiny cavities and crosslinks, suggesting that Lipiodol, which has difficulty mixing with blood, may have existed as oil droplets in the cavities. This reaction time may have been sufficient for the mixed Lipiodol to remain as fine oil droplets. In contrast, for NLE221, low-molecular-weight polymers that had already been formed may have quickly initiated polymerization following contact with blood, leading to immediate folding due to polymerization shrinkage and thus forming a folded-sheet structure through repeated folding [13]. Compared to the honeycomb-like structure, the folded-sheet structure has a smaller contact area with the catheters and vascular intima, which may have also contributed to the decrease in the adhesive strength of the NLE.

NLE230② sample in this study exhibited a partial mix of honeycomb-like and folded-sheet structures. If these differences were caused by variations in the reaction speed, it is not inconsistent for the folded-sheet structure to be present in the NLE230 polymer if the reaction speed is faster for some reason. It is considered that the folded-sheet structure dominated in NLE150 because the low NBCA ratio likely caused the reaction to proceed rapidly due to the increased contact with a large number of initiators upon injection. Additionally, when comparing NLE150 and NLE141, the density of the polymerized material in NLE141 was lower, forming a more fragile polymer. The weak bonding between polymer chains led to partial breakage during the degreasing and dehydration process. Even with the same NBCA concentration, polymers formed with the addition of ethanol may be more fragile, particularly in embolization situations where blood flow is present, which could be a point of concern. Additionally, the low adhesive property of lower-concentration NLE may also be due to the fragility of its polymerized material.

This study has some limitations. First, it is an in vitro study. Second, the effects of factors such as blood flow, inflammation, platelets, and coagulation factors are not reflected, and the defatting and dehydration process has been included. Third, the sample size is small. At last, specimens were each created on different days. While the work was conducted in the angiography room, where the room temperature was relatively stable, the environment did not replicate body temperature (36 °C).

Although many aspects of the reaction mechanism remain unclear, it has been suggested that under certain conditions, there are structural differences between NL and NLE. Furthermore, NLE polymers may be more fragile than NL polymers. In order to ensure the safe and effective use of NLE, further investigation and validation of its characteristics will be necessary.
